# Differences in Explicit Stereotype Activation among Social Groups Based on the Stereotype Content Model: Behavioral and Electrophysiological Evidence in Chinese Sample

**DOI:** 10.3390/brainsci10121001

**Published:** 2020-12-17

**Authors:** Yaping Yang, Katherine R. G. White, Xinfang Fan, Qiang Xu, Qing-Wei Chen

**Affiliations:** 1 Department of Psychology, Ningbo University, Ningbo 315211, China; yangyaping@nbu.edu.cn (Y.Y.); 1911041210@nbu.edu.cn (X.F.); xuqiang1@nbu.edu.cn (Q.X.); 2 Department of Psychological Science, Kennesaw State University, Kennesaw, GA 30144, USA; kwhit162@kennesaw.edu; 3 Lab of Light and Physiopsychological Health, National Center for International Research on Green Optoelectronics, South China Normal University, Guangzhou 510006, China; 4 Guangdong Provincial Key Laboratory of Optical Information Materials and Technology & Institute of Electronic Paper Displays, South China Academy of Advanced Optoelectronics, South China Normal University, Guangzhou 510006, China; 5 School of Psychology, South China Normal University, Guangzhou 510631, China

**Keywords:** stereotype activation, stereotype content model, N400, LPC, P2, event-related potentials, reverse priming effect

## Abstract

The stereotype content model (SCM; Fiske, Cuddy, Glick and Xu, 2002) identifies four basic categories of stereotyped social groups: high warmth-high competence (HW-HC), high warmth-low competence (HW-LC), low warmth-high competence (LW-HC), and low warmth-low competence (LW-LC). However, many of these groups have not been directly examined in stereotype activation research. The purpose of the present research was to extend stereotype activation research to groups that more fully represent those identified under the SCM. Employing explicit sequential priming task, participants responded to prime-target stimulus pairs that were either congruent or incongruent with stereotypes of social groups from all four SCM quadrants in two studies in the current investigation. Study 1 was to determine the behavioral pattern of explicit stereotype activation among four quadrants (the sample included 60 Chinese undergraduate students, 51%—female). Study 2 further employed event-related brain potentials (ERPs) technique to track the time course and electrophysiological underpinnings of explicit stereotype activation (the sample included 22 right-handed Chinese undergraduate students, 76%—female). In Study 1, participants responded more quickly and accurately on stereotype congruent trials than incongruent trials for all social groups except LW-LC groups. This reverse priming effect on LW-LC social groups in RTs was also replicated in Study 2. ERPs findings further showed that incongruent targets elicited larger N400 amplitudes than congruent targets for all four SCM quadrants. Moreover, congruent targets elicited larger P2 than incongruent targets, but only found for the LW-LC social groups. In addition, congruent targets elicited larger amplitudes of late positive component than incongruent targets for the low warmth (LW-LC and LW-HC) groups. Together, these results highlight the unique processing that LW-LC groups receive throughout the cognitive stream, ultimately manifesting in distinctive behavioral responses. Unconscious activation of egalitarian goals, disgust, and distrust accounts are discussed.

## 1. Introduction

In an effort to process and simplify a complex social world, people often rely on mental shortcuts and heuristics as they navigate their daily lives [1,2]. Stereotypes represent one of these mental shortcuts, and while their use can provide a sense of order and coherence, they can just as easily lead to misunderstandings and friction in one’s social environment. The activation of stereotypes and their resulting impact on human behavior are seen as a ubiquitous part of being human, thus they have been the subject of intense research scrutiny for over half a century. Starting in the 1980s, researchers adapted response time paradigms to study stereotype activation (e.g., [3,4,5,6]). More recently, various neuroscience techniques have been used to further elucidate the cognitive processes involved in stereotype activation (see reviews [7,8,9]). This line of research has revealed a great deal about stereotype activation and the conditions under which it occurs (for review, see [10]), but the vast majority has focused on only one or two social groups (e.g., racial groups, gender groups). According to the stereotype content model (SCM [11,12]), there are many other groups in society that are consistently stereotyped, and these groups also require empirical attention. The primary purpose of the present research was therefore to investigate patterns of stereotype activation in both response times and neural activity for a wide range of social groups identified under the SCM. 

### 1.1. The Stereotype Content Model (SCM) and BIAS Map

The SCM [11,13] organizes social and cultural groups along two universal and fundamental dimensions: perceived warmth and perceived competence. Warmth refers to the intent to help or harm others, and warmth judgments are dictated primarily by perceptions of competition (competing groups are perceived as less warm). Competence refers more specifically to the ability to help or cause harm, and judgments are based more on the perceived status of the group in society (lower status groups are perceived as less competent). Social and cultural groups in society are perceived as low or high on each of these dimensions, resulting in their placement in one of four different quadrants of the model. Some groups are perceived as high on both warmth and competence (HW-HC). These are typically groups that participants perceive as part of their in-group or are held in relatively universal high regard within society. On the other end of the spectrum, some groups are perceived as low on both warmth and competence (LW-LC). These groups are held in relatively universal low regard and include groups such as the homeless and drug addicts. Finally, there are groups in society that have mixed stereotype content, meaning they are perceived as high on one dimension, but low on the other. For example, some groups are perceived as high on warmth but low on competence (HW-LC) (e.g., elderly people, the disabled). Other groups are perceived as low on warmth but high on competence (LW-HC) (e.g., rich people). Research has confirmed that SCM patterns generalize across time, level of analysis, and measures (see reviews [14,15]). Additionally, the SCM has been validated in multiple countries [16,17,18,19]. To further develop the SCM, BIAS (behaviors from intergroup affect and stereotype) map [12,20] was proposed to capture the affect and action tendencies associated with stereotype. Besides the application in intergroup situations, SCM and BIAS map could also be employed in interpersonal perception [21,22,23].

### 1.2. Low Warmth—Low Competence (LW-LC) Social Groups

Among the four quadrants of the SCM, groups in the LW-LC quadrant might be processed differently from groups in the other three quadrants. According to the SCM and BIAS map, LW-LC social groups elicit contempt and disgust along with active harm behavioral tendencies (e.g., attacking or harassing) [11,12,13,20]. Social groups in the LW-LC (vs. HW-HC, HW-LC, LW-HC) quadrant fail to elicit brain activity associated with human social processing (medial pre-frontal cortex, mPFC) and instead elicit activity associated with disgust (insula and amygdala) [24,25] but these findings have not been replicated by others. In other words, it has been argued that members of social groups in the LW-LC quadrant are more likely to be dehumanized relative to social groups in the other three quadrants (see [26,27] for a more complete discussion). Other research demonstrated that brain regions associated with the emotion of disgust (the left anterior insula) are more active when thinking about the homeless (LW-LC) than the elderly (HW-LC) [28]. Compared with other three quadrants, participants were more willing to sacrifice LW-LC group members in moral dilemmas [29]. Moreover, desirable events were anticipated to occur significantly more frequently than undesirable events for HW-HC and HW-LC group members while this social optimism biases were reversed for LW-LC groups [30,31,32]. Compared to HW-HC and HW-LC groups, the temporoparietal junction and right anterior temporal lobe were recruited when evaluating LW-HC and LW-LC groups, while thinking about LW-LC groups additionally involved the dorsomedial frontal cortex (stereotypical thinking and disgust), inferior frontal cortex (stereotypical thinking), and anterior insula (disgust and repulsion) [31].

### 1.3. Response Pattern of Stereotype Activation

Behavioral research on stereotype activation has relied heavily on priming and response time techniques (e.g., [33,34,35,36]). A common paradigm used in stereotype research is the sequential priming paradigm [37,38]. In this paradigm, participants view trials that each contains two stimuli (prime and target) with the meaning congruency between the stimuli varied across trials. For example, in stereotype priming, a congruent stimulus pair would be the prime “men” followed by the stereotypically congruent target “strong”. An incongruent stimulus pair would be the prime “women” followed by the stereotypically incongruent target “strong”. If participants respond more quickly to a target preceded by a stereotypically congruent prime than those preceded by a stereotypically incongruent prime, this is interpreted as evidence of stereotype priming/activation. The sequential priming paradigm has been used to examine stereotypes for a variety of different social groups [37,38] (such as gender [3,39,40,41,42,43,44,45,46,47,48,49,50,51,52,53,54], and race [52,53,54,55,56,57,58,59,60,61,62,63,64,65,66,67]) and the priming effect was repeatedly reported [1,68,69], but we are not aware of any research that examined groups from all SCM quadrants simultaneously and compared observed priming effects among them. Thus, the purpose of Study 1 in the present research was to investigate the pattern of stereotype activation in behavioral response for social groups that fall into the four different quadrants of the SCM. In line with previous research, we expected to observe lower accuracy and slower response times on stereotype incongruent trials than stereotype congruent trials, and that this pattern would be observed for social groups from all four SCM quadrants.

### 1.4. Electrophysiological Pattern of Stereotype Activation

Stereotype activation occurs rapidly and involves multiple cognitive processes [8,9,43,70,71]. Due to high temporal resolution (milliseconds), event-related potential (ERP) is optimal for studying the progress of stereotype activation as it unfolds in real-time [72,73]. The various ERP components are also sensitive to different types of cognitive processes, enabling researchers to disentangle the presence and impact of these processes. Thus, ERPs are better able to examine the time course of stereotype activation, and the cognitive processes that contribute to this activation, than either response times or fMRI data. Similar to response times research, however, ERP research on stereotypes and stereotype activation has focused on a limited number of social groups (e.g., men/women, black/white) [40,41,67,74,75]. The utility of ERPs to study all types of SCM groups therefore remains untested. Study 2 in the present research had two primary goals: (1) extend neuropsychological investigations of the SCM by using ERPs to elucidate the cognitive processing time-course for multiple social groups, and (2) extend previous ERP research on stereotype activation by identifying potential differences among groups in the four SCM quadrants. These goals were accomplished by examining ERP components at different time points in the information-processing stream. 

#### 1.4.1. Early ERP Component: P2

The P2 component has previously been connected with stereotype processing. While the P2 component appears to be modulated by a variety of cognitive tasks and conditions, it is generally thought to also reflect mechanisms of selective attention [76]. Previous research has found the P2 component to be particularly sensitive to race and stereotypes. Specifically, larger P2 amplitudes were elicited by pictures of black faces (than white faces) among white participants (e.g., [77,78,79,80]), and larger P2 amplitudes have been observed on race stereotype congruent trials than incongruent trials in a sequential priming paradigm [67]. These results still need to be replicated for other stereotyped social groups in society. Given the uniquely stigmatized status of LW-LC groups, we expected these groups to attract greater attention early in the processing stream, which would be reflected in larger P2 amplitudes on stereotype-congruent trials for these groups. 

#### 1.4.2. Later ERP Components: N400 and LPC

Two later ERP components have been examined in research on stereotypes and stereotype activation—the N400 and the late positive component (LPC). The N400 component is a negative deflection that peaks around 400 milliseconds following stimulus exposure. It is thought to reflect the influence of both bottom-up and top-down neural processes in efforts to construct meaning, drawing on information from the multimodal long-term memory system [81]. The amplitude of the N400 is particularly responsive to the ease of integrating information into the preceding context, with larger amplitudes elicited when integration is more difficult. For example, larger N400 amplitudes would be elicited by the word “jelly” if preceded by the word “shoe” than if preceded by the word “grape” [82]. One potential explanation for these results is that exposure to the first word (“grape”) results in the activation of long-term memory information related to this concept as the system attempts to reconstruct the meaning of this concept. If the second word is related to the concept of “grape” (as would be the case for the word “jelly”), then the preactivation of this information would facilitate processing of the second word. Thus, differences in N400 amplitudes may reflect differences in the activation of multimodal information in long-term memory during the meaning-construction process [81]. 

Recent research extended earlier N400 findings to stereotypes, and consistently found the N400 to be sensitive to stereotype incongruities (e.g., [40,41,67,75,83]). This was first demonstrated by White and colleagues (2009) [40], who presented participants with prime-target stimulus pairs which were congruent or incongruent with gender stereotypes. In this study, stereotype incongruent trials (e.g., MEN: NURSE) elicited significantly larger N400 amplitudes than stereotype congruent trials (e.g., WOMEN: NURSE). Later studies replicated these results for gender stereotypes in a non-Western culture (i.e., Chinese culture [39,41,42,43]) and for other stereotype dimensions, such as race [67] and migrant workers [83]. This suggests that the N400 ERP component is sensitive to stereotype incongruities and may reflect differences in stereotype activation. However, whether these results could be replicated and generalized in social groups that represent all four quadrants of the SCM still remains unclear. Aligned with previous research, we expected that stereotype-incongruent targets would elicit larger N400 amplitudes than congruent targets for social groups in all four quadrants of the SCM. 

Evidence for the sensitivity of the LPC to stereotypes is more mixed. The LPC is a positive deflection with typical onset around 300 milliseconds, extending for several hundred additional milliseconds. In one early study, participants read sentences that violated definitional (e.g., the mailman took a shower after she got home) or stereotypical (e.g., our aerobics instructor gave himself a break) noun-pronoun agreement [84]. Relative to sentences without a noun-pronoun violation, LPC amplitudes were enhanced for both definitionally and stereotypically incongruent sentences, suggesting that the LPC might be sensitive to stereotype incongruities. Other studies also found the LPC to be sensitive to pronoun incongruities within the context of social and nonsocial sentences [85,86]. In contrast to these results, the LPC was not modulated by stereotype incongruities in studies that employed a sequential priming paradigm (i.e., presented participants with word pairs rather than sentences) (e.g., [40,41,67]). Given these latter results, we did not expect stereotype congruity to modulate LPC amplitudes in Study 2.

### 1.5. Current Research

In the current research, we conducted two studies to explore the similarities and differences on explicit stereotype activation for HW-HC, HW-LC, LW-HC and LW-LC social groups based on the framework of SCM. Employing sequential priming paradigm, we investigated the behavioral pattern of explicit stereotype activation in Study 1 and further examined the electrophysiological pattern of explicit stereotype activation among four quadrants by using ERPs in Study 2.

## 2. Study 1: The Behavioral Pattern of Explicit Stereotype Activation for HW-HC, HW-LC, LW-HC and LW-LC Social Groups

### 2.1. Methods

#### 2.1.1. Participants

Sixty Chinese undergraduate students (31 female, M = 21.5 years, range 19–25 years) participated in the study in exchange for 10 RMB. All participants signed an informed consent form approved by the Academic Ethical Committee of Ningbo University. Data from two participants (1 female and 1 male) were excluded due to excessive incorrect trials (greater than 30%), leaving a final sample of 58 participants. 

#### 2.1.2. Design

Group (HW-HC, HW-LC, LW-HC, LW-LC) × congruency (congruent, incongruent) within-subject design was applied in the current study.

#### 2.1.3. Stimuli

Stimuli were Chinese word pairs consisting of a prime word followed by a target word. Prime words were 24 social group labels, which were retrieved from Yang et al. (2019) [87], which were validated in Ji et al. (2020) [88] (see Appendix A). These social groups were divided into four categories, according to the two SCM dimensions of warmth and competence. Specifically, the six high warmth-high competence (HW-HC) groups were soldiers, firemen, professors, psychotherapists, air hostesses, and yoga instructors. The six high warmth-low competence (HW-LC) groups included elderly, farmers, housewives, migrant workers, left-behind children, and cleaning workers. The six low warmth-high competence (LW-HC) groups were businessmen, overseas returnees, government workers, government officials, stars in showbiz, and rich. Finally, the six low warmth-low competence (LW-LC) groups included criminals, unemployed, beggars, drug addicts, terrorists, and urban management officers. 

Target stimuli were words stereotypically associated with one of the social groups. Target words were also retrieved from Yang et al. (2019) [87], which were selected through the classical method by Katz and Braly (1933) [89] (see [88] for details). For each quadrant of SCM, the 30 most stereotypic words were selected, totaling 120 target words (see Appendix A).

These 120 words were used to form both congruent and incongruent trials. When paired with their matching social group, the words formed congruent trials. Incongruent trials were then created by pairing each target word with a social group that was as different in stereotypes as possible. This resulted in 30 congruent and 30 incongruent target words for each social group category (see Appendix A). Thus, eight experimental conditions were established: stereotype congruent vs. incongruent for the HW-HC social group category, stereotype congruent vs. incongruent for the HW-LC social group category, stereotype congruent vs. incongruent for the LW-HC social group category, and stereotype congruent vs. incongruent for the LW-LC social group category. In total, there were 120 congruent prime-target trials and 120 incongruent prime-target trials. Each prime-target word pair was presented twice, thus there were 480 trials in the experiment. 

**Figure 1:**
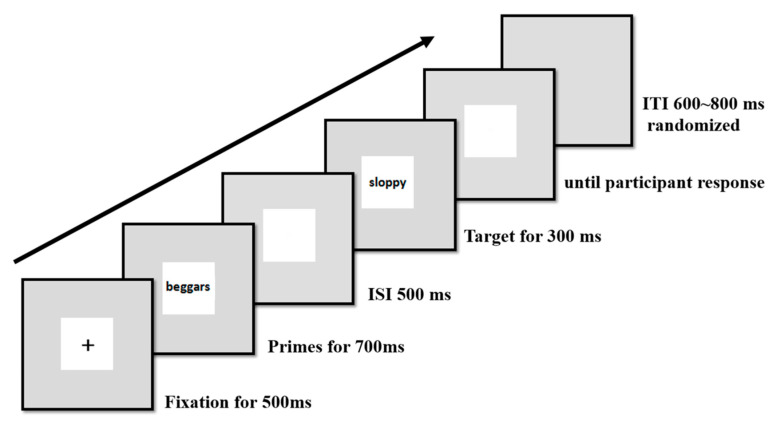
Example of the sequences of one trial. ITI: inter-trial interval; ISI: inter-stimulus interval.

All primes and targets (Chinese characters) were presented centrally on a white background in 60-point, black, boldface font. All stimuli were presented in the center of a 17 in. LCD (Liquid Crystal Display) monitor with a silver-gray background (resolution: 1024 × 768, refresh rate: 60 Hz) using E-Prime 2.0 (see Figure 1). The stimuli, seen from a distance of 120 cm, occupied a visual angle of 6.06° × 6.06°.

#### 2.1.4. Procedure

Upon arriving at the lab, participants read and signed an informed consent form. Each participant was escorted to a dimly lit, sound attenuated testing room and seated in a comfortable chair approximately 120 cm in front of a computer monitor. Participants were instructed to watch the center of the screen where a series of word pairs would be presented. It was explained that each trial would begin with a fixation point, then they would see a social group label (e.g., bagger), and the second word would sometimes be associated with the preceding social group (i.e., sloppy) and other times not associated with the preceding social group (i.e., pretty). Participants were instructed to verify as quickly and as accurately as possible whether or not the second word matched the priming word according to cultural stereotypes by pressing the “CONGRUENT” button on the response keyboard for matching words and the “INCONGRUENT” button on the response keyboard for non-matching words. The “CONGRUENT” and “INCONGRUENT” labels were pasted on the “Q” and “P” keys of the keyboard before the experiment and were counterbalanced across participants. 

As presented in Figure 1, each trial began with the appearance of a fixation point for 500 ms. The fixation point was then replaced with a prime stimulus, which remained on the screen for 700 ms. After an inter-stimulus interval (ISI) of 500 ms (SOA (Stimulus Onset Asynchrony) = 1200 ms), the target stimulus was presented for 300 ms. The screen then went blank and remained blank until the participant responded. The inter-trial interval (ITI) was randomized between 600 and 800 ms. 

Participants completed a total of 528 trials, 480 of which constituted the experimental trials (presented in 6 blocks of 80 trials). The first 48 trials were presented as practice trials to familiarize participants with the procedure. Six social groups (policemen, otaku, Tibetan girls, football players, models, and the mentally disabled) were used as practice primes. The 480 experimental trials were presented after these practice trials. Stimuli used in practice trials did not appear in the experimental trials and only data from the experimental trials was analyzed. Participants took a break (about 3 to 4 min in length) after each block of 80 trials.

#### 2.1.5. Data Analyses

Accuracy rates and response times (RTs) were analyzed using two-way repeated measures ANOVAs, with both groups (HW-HC, HW-LC, LW-HC, LW-LC) and congruency (congruent, incongruent) as within-subject factors. For each participant, incorrect responses and response times that deviated ±2 SD from each condition were removed before formal analysis [90]. Statistical analyses were conducted using SPSS 13.0. When the assumption of sphericity was violated, the Greenhouse–Geisser (G–G) adjustment, with the corrected significant levels and partial eta-squared (*η*^2^) effect-size statistic, are reported. For simplicity, the uncorrected degrees of freedom are presented along with the G–G epsilon (ε). Post hoc comparisons were conducted using a Bonferroni correction to control for inflated family-wise error. 

### 2.2. Results

#### 2.2.1. Accuracy

**Figure 2:**
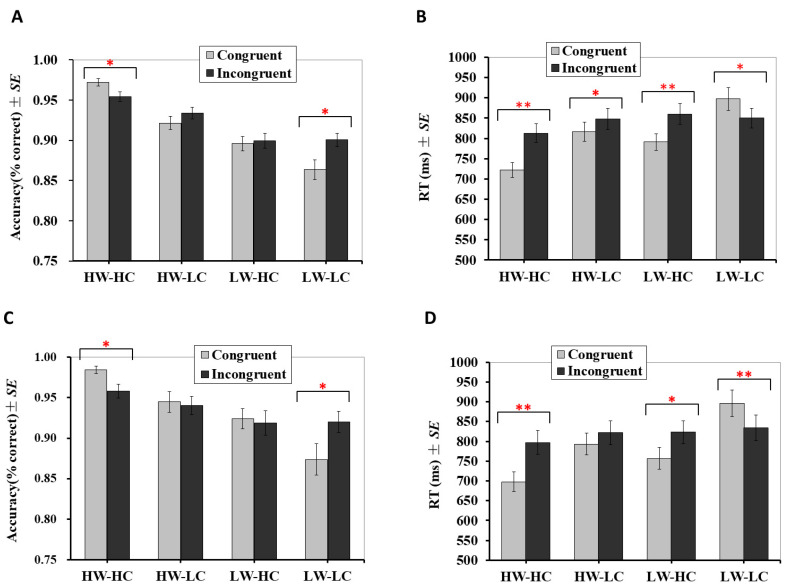
Behavioral results for Study 1 and Study 2. (**A**) Accuracy in Study 1; (**B**) mean reaction times in Study 1 (*n* = 58); (**C**) accuracy in Study 2; (**D**) mean reaction times in Study 2 (*n* = 19). Error bars indicate ± 1 standard error of the mean. RT: reaction time. SE: standard error. ** *p* < 0.01, * *p* < 0.05.

The analysis revealed a significant main effect for group, F (3, 171) = 93.58, *p* < 0.001, partial *η*^2^ = 0.62, but not for congruency, F (1, 57) = 1.24, *p* = 0.270, partial *η*^2^ = 0.02. The main effect for group was qualified by a significant interaction with congruency, F (3, 171) = 7.22, *p* < 0.001, partial *η*^2^ = 0.11. As presented in Figure 2A, simple effect analyses for the HW-HC group showed that accuracy on trials with congruent targets (M = 0.972, SE = 0.005) was significantly higher than for trials with incongruent targets (M = 0.955, SE = 0.006), *p* = 0.002. The difference in accuracy between congruent and incongruent trials was also significant for the LW-LC group, but in the opposite direction; accuracy on trials with congruent targets (M = 0.864, SE = 0.012) was significantly lower than trials with incongruent targets (M = 0.901, SE = 0.008), *p* = 0.009. There was no significant difference in accuracy between trials with congruent and incongruent targets for the HW-LC (for congruent targets: M = 0.922, SE = 0.008; for incongruent targets: M = 0.934, SE = 0.007; *p* = 0.154) or the LW-HC groups (for congruent targets: M = 0.896, SE = 0.009; for incongruent targets: M = 0.900, SE = 0.009; *p* = 0.781).

#### 2.2.2. Response Times

Analysis of RTs revealed significant main effects for both *Group*, F (3, 171) = 66.67, *p* < 0.001, partial *η*^2^ = 0.54, and congruency, F (1, 57) = 13.34, *p* = 0.001, partial *η*^2^ = 0.19. The interaction between group and congruency was also significant, F (3, 171) = 30.41, *p* < 0.001, partial *η*^2^ = 0.35. As presented in Figure 2B, participants responded significantly faster to congruent targets than incongruent targets for the HW-HC (for congruent targets: M = 722 ms, SE =18; for incongruent targets: M = 813 ms, SE = 23; *p* < 0.001), HW-LC (for congruent targets: M = 817 ms, SE = 23; for incongruent targets: M = 848 ms, SE = 27; *p* = 0.031), and LW-HC groups (for congruent targets: M = 791 ms, SE = 20; for incongruent targets: M = 860 ms, SE = 26; *p* < 0.001). Conversely, participants responded significantly slower to congruent targets (M = 898 ms, SE = 28) than to incongruent targets for the LW-LC group (M = 850 ms, SE = 24), *p* = 0.003. 

### 2.3. Discussion

Using a sequential priming paradigm, Study 1 examined the behavioral pattern of stereotype priming on accuracy rates and response times for the four quadrants (HW-HC, HW-LC, LW-LC, and LW-HC) identified under the framework of SCM. The typical stereotype priming pattern was observed (especially in RTs) for the HW-HC, HW-LC and LW-HC social groups. That is, congruent targets elicited significantly faster responses than incongruent targets for these social groups, replicating previous studies [3,6,40,42,47]. More importantly, a reverse stereotype priming pattern for LW-LC groups was revealed in Study 1. That is, congruent targets elicited significantly slower responses than incongruent targets, which indicated the robustness of reverse priming effect for LW-LC social groups.

Overall, the results from Study 1 replicate the typical stereotype priming pattern for HW-HC, HW-LC and LW-HC social groups, but reveal a decidedly reverse stereotype priming pattern for LW-LC social groups. Accuracy rates and response times may not be sensitive enough to adequately probe stereotype priming results, though. Behavioral RTs represent the outcome of a set of cognitive processes, and thus are limited to measuring a single conscious outcome of these processes [91]. For example, response times in sequential priming paradigms may reflect the influence of memory activation and response competition [92]. Distinguishing among these processes in behavioral results can be done using specific analyses and models (e.g., [93,94,95]), but an alternative approach is to measure stereotype activation more directly with event-related potentials (ERPs). This approach was adopted in Study 2, which used both behavioral and ERP measures to further examine patterns of stereotype activation. 

## 3. Study 2: The Electrophysiological Pattern of Explicit Stereotype Activation for HW-HC, HW-LC, LW-HC and LW-LC Social Groups

The purpose of Study 2 was to explore potential neuropsychological differences in stereotype activation patterns among the four SCM clusters. Both early (P2) and later (N400, LPC) ERP components were examined to determine if stereotype activation patterns differ among four SCM quadrants, both in magnitude of activation and timing. The N400 ERP component was of primary importance, given that previous research has found it to be a reliable measure of stereotype activation [39,40,41,42,67,83], and it reflects a post-perceptual processing stage that involves the dynamic reconstruction of information from long-term memory [81]. In this way, Study 2 aimed to demonstrate the utility of the N400 ERP component as a measure of stereotype activation for a wide range of social groups identified under the framework of SCM. In addition to the N400, we also examined early ERP component (P2) at fronto-central regions to capture early perceptual processing stages and the late positive component (LPC), which reflects a later, decision processing stage.

Overall, we expected to replicate the results of Study 1, that is, a typical stereotype priming effect in response times for the HW-HC, HW-LC, and LW-HC groups, and reverse priming effect for the LW-LC groups. For the N400, we expected stereotype incongruent trials to elicit larger N400 amplitudes than stereotype congruent trials for all four SCM group clusters, but that this effect would be especially pronounced for the LW-LC social group cluster. Due to the attention-grabbing nature of the LW-LC groups, we also expected these social groups to elicit larger P2 amplitudes than the other SCM clusters. No differences were expected for any of the remaining ERP components. 

### 3.1. Methods

#### 3.1.1. Participants

Twenty-two right-handed, Chinese undergraduate students (16 female, M = 21.9 years, range 19–25 years) participated in the study in exchange for 30 RMB. All participants reported normal or corrected-to-normal vision, had no history of current or past neurological or psychiatric illness, and took no medications known to affect the central nervous system. All signed an informed consent form approved by the Academic Ethical Committee of Ningbo University. Data from three participants (1 female and 2 male) were excluded due to excessive artifacts, leaving a final sample of 19 participants. 

#### 3.1.2. Stimuli and Procedure 

The stimuli used in Study 2 were the same as those used in Study 1. The procedures used were also identical, except that participants were prepared for electroencephalogram (EEG) recording before the formal experiment.

#### 3.1.3. EEG Recording

EEG data was recorded continuously using an electrode cap with 64 Ag/AgCl electrodes mounted according to the extended international 10–20 system. Electro-oculargram (EOG) data was recorded via two pairs of electrodes placed on the bilateral external canthi and left supraorbital and infraorbital areas to monitor eye movements and blinks. Both EEG and EOG were sampled at 500 Hz, with a bandpass of 0.05–100 Hz using NeuroScan Synamps2 Amplifier. The left mastoid served as a reference during recording and the right mastoid served as a recording electrode. Electrode impedances were kept below 5 kΩ. 

NeuroScan 4.3 software (Compumedics Neuroscan, Charlotte, NC, USA) was used to analyze the data offline. The data was re-referenced offline to the algebraic average of the left and right mastoids. The raw EEG data were manually previewed to remove artifacts after merging with the behavioral data. Then an automatic eye-movement correction program corrected vertical eye movements and blinks [96]. Afterwards, the EEG was segmented in epochs of 1200 ms beginning 200 ms prior to the target stimulus onset and each epoch in all electrodes was then corrected to a 200 ms baseline. Segments contaminated with artifacts exceeding an amplitude of ±80 μV were automatically rejected from the averaging (9.7% artifacts were rejected). EEG data from correct trials were averaged separately for each of the eight conditions. After this procedure, averaged ERPs included at least 48 trials for each trial type. Finally, the averaged ERPs were low-pass filtered at 30 Hz (24 dB/Oct ave).

#### 3.1.4. Data Analyses

Behavioral data were analyzed using the same method in Study 1. For ERPs data, mean amplitude of each ERP component (P2, N400, LPC) was subjected to two-way repeated measures ANOVA with both group (HW-HC, HW-LC, LW-HC, LW-LC) and congruency (congruent, incongruent) as within-subject factors. The analyses were conducted on ERP mean amplitudes of clusters of electrodes (regions of interest, ROI). The measurement windows and electrodes to be included in the ROIs were determined by visual inspection of the ERP waveforms and literature-based hypotheses (for exact time-windows and ROIs of each component please see the corresponding section in the results) [97]. All post-hoc tests were Bonferroni corrected for multiple testing. If necessary, the degrees of freedom were corrected using the Greenhouse–Geisser epsilon.

### 3.2. Results

#### 3.2.1. Behavioral Results

***Accuracy*.** Accuracy results mirrored those from Study 1. That is, the main effect was significant for group, F (3, 54) = 23.55, *p* < 0.001, partial *η*^2^ = 0.57, but not for congruency, F (1, 18) = 0.06, *p* = 0.809, partial *η*^2^ = 0.003. Additionally, the main effect for group was qualified by a significant interaction with congruency, F (3, 54) = 4.88, *p* = 0.005, partial *η*^2^ = 0.21. As presented in Figure 2C, simple effect analyses for the HW-HC quadrant revealed that accuracy on congruent trials (M = 0.984, SE = 0.005) was significantly higher than incongruent trials (M = 0.958, SE = 0.008), *p* = 0.002. The difference in accuracy between congruent and incongruent trials was also significant for the LW-LC quadrant, but in the opposite direction; accuracy on congruent trials (M = 0.874, SE = 0.019) was significantly lower than incongruent trials (M = 0.920, SE = 0.013), *p* = 0.012. There was no significant difference in accuracy between congruent and incongruent trials for the HW-LC (for congruent targets: M = 0.945, SE = 0.013; for incongruent targets: M = 0.940, SE = 0.011; *p* = 0.762) or LW-HC groups (for congruent targets: M = 0.924, SE = 0.012; for incongruent targets: M = 0.918, SE = 0.015; *p* = 0.819). 

***Response times*.** Similar to Study 1, for HW-HC, LW-LC and LW-HC groups, analysis of RTs revealed significant main effects for both group, F (3, 54) = 49.44, *p* < 0.001, partial *η*^2^ = 0.73, and congruency, F (1, 18) = 9.45, *p* = 0.007, partial *η*^2^ = 0.34. The interaction between group and congruency was also significant, F (3, 54) = 25.38, *p* < 0.001, partial *η*^2^ = 0.59. Simple effect analyses showed that, as presented in Figure 2D, participants responded significantly faster to congruent targets than incongruent targets for the HW-HC (for congruent targets: M = 698 ms, SE = 25; for incongruent targets: M = 797 ms, SE = 31; *p* < 0.001) and LW-HC groups (for congruent targets: M = 757 ms, SE = 28; for incongruent targets: M = 823 ms, SE = 28; *p* < 0.001). Although participants also responded faster to congruent targets (M = 793 ms, SE = 28) than incongruent targets (M = 822 ms, SE = 30) for the HW-LC groups, the difference did not reach a statistically significant level (*p* = 0.122). Conversely, participants responded significantly slower to congruent targets (M = 896 ms, SE = 34) than to incongruent targets (M = 834 ms, SE = 32) for the LW-LC group (*p* = 0.001). 

#### 3.2.2. ERPs Results

**Figure 3:**
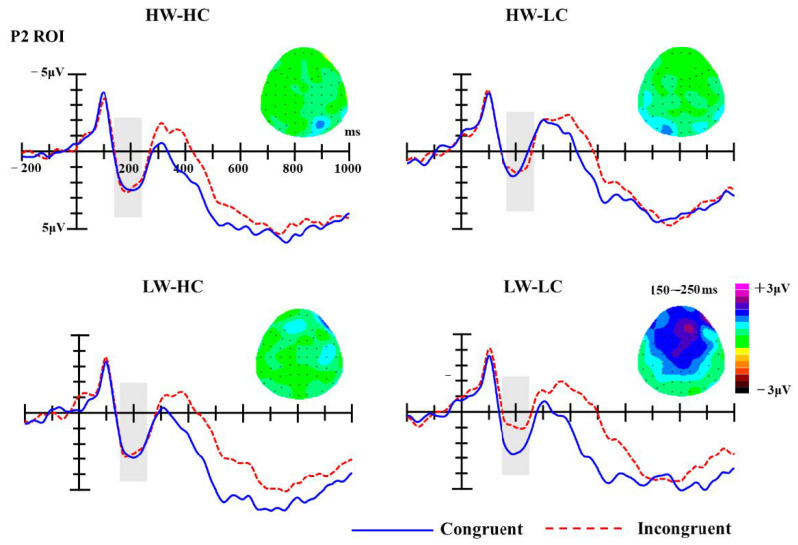
The grand-average event-related brain potentials (ERPs) at selected regions of interest (ROI) electrodes (Fz, FCz, Cz); the shaded areas mark P2 components (150–250 ms). Topographies of P2 difference waves (incongruent minus congruent trials) for four stereotype content module (SCM) quadrants.

***P2***. For the P2 component, the ROI included 3 central electrodes (Fz, FCz, Cz) in the 150–250 ms time-window. Amplitudes were averaged across all electrodes within the ROI before submitting them to statistical analysis. Analysis of P2 mean amplitudes revealed significant main effect of group, F (3, 54) = 10.14, *p* < 0.001, partial *η*^2^ = 0.36, but the main effect of congruency was not, F (1, 18) = 1.47, *p* = 0.241, partial *η*^2^ = 0.08. The interaction between group and congruency was significant, F (3, 54) = 2.78, *p* = 0.050, partial *η*^2^ = 0.13. Simple effect analyses revealed that the P2 amplitudes elicited by congruent targets (M = 2.56 μV) were significantly more positive than those elicited by incongruent targets (M = 1.20 μV; *p* = 0.002), but only for the LW-LC social groups (see Figure 3). 

***N400*.** Similar to the statistical analyses of the P2 component, analyses of the N400 component were conducted on mean ERP amplitudes of the given ROI (FC1, FCz, FC2, C1, Cz, C2, CP1, CPz, CP2) in the 300–500 ms time-window. Analysis of N400 mean amplitudes revealed a significant main effect of group, F (3, 54) = 9.23, *p* < 0.001, partial *η*^2^ = 0.34, as well as a significant main effect of congruency, F (1, 18) = 164.93, *p* < 0.001, partial *η*^2^ = 0.90. Specifically, the N400 amplitude elicited by incongruent targets (M = −0.69 μV) was significantly more negative than that elicited by congruent targets (M = 1.74 μV). Moreover, the interaction between group and congruency was also significant, F (3, 54) = 2.79, *p* = 0.049, partial *η*^2^ = 0.13.

**Figure 4:**
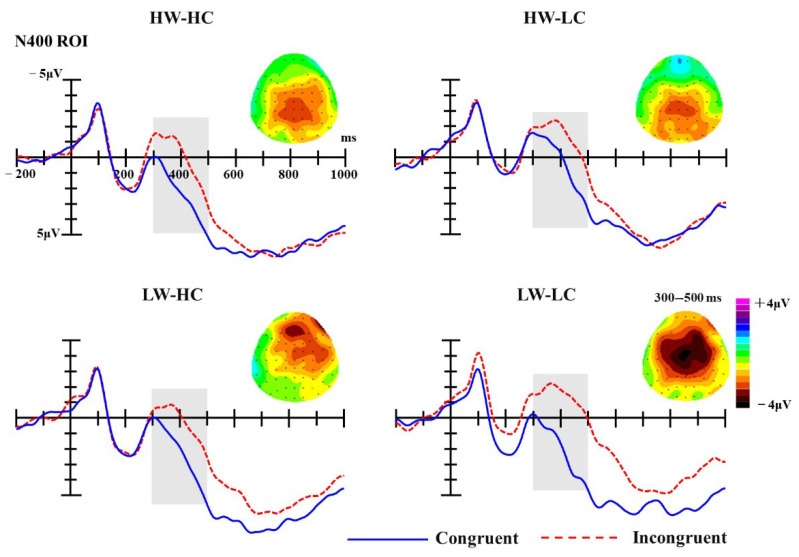
The grand-average ERPs at selected ROI electrodes (FC1, FCz, FC2, C1, Cz, C2, CP1, CPz, CP2); the shaded areas mark N400 components (300–500 ms). Topographies of N400 difference waves (incongruent minus congruent trials) for four SCM quadrants.

Simple effect analyses for the group × congruency interaction confirmed the main effect of congruency. As illustrated in Figure 4, the N400 amplitudes elicited by incongruent targets was significantly more negative than those elicited by congruent targets, for all four quadrants (for HW-HC quadrant: M _incongruent_ = −0.06 μV, M _congruent_ = 2.36 μV, MD _incongruent-congruent_ = −2.42 μV, SE = 0.37, *p* < 0.001; for HW-LC quadrant: M _incongruent_ = −1.43 μV, M _congruent_ = 0.32 μV, MD _incongruent-congruent_ = −1.75 μV, SE = 0.37, *p* < 0.001; for LW-HC quadrant: M _incongruent_ = 0.20 μV, M _congruent_ = 2.28 μV; MD _incongruent-congruent_ = −2.08 μV, SE = 0.59, *p* = 0.002; for LW-LC quadrant: M _incongruent_ = −1.48 μV, M _congruent_ = 2.02 μV, MD _incongruent-congruent_ = −3.49 μV, SE = 0.36, *p* < 0.001). However, as presented in the Figure 4, this tendency seems more obvious for the LW-LC quadrant.

To further test the discrepancies on N400 component induced by different warmth-competence social groups, difference waves were calculated (subtracted the N400 amplitude elicited by congruent trials from the N400 amplitude elicited by incongruent trials within each social group) and analyzed using a one-way repeated measure ANOVA with group (HW-HC, HW-LC, LW-LC, LW-HC) as a within-subject factor. Results revealed a significant main effect for group, F (3, 54) = 2.79, *p* = 0.049, partial *η*^2^ = 0.13. Multiple comparisons showed that the N400 difference waves elicited by LW-LC social groups were the largest (M = −3.49 μV) and were significantly more negative than those elicited by the HW-LC social groups (M = −1.75 μV; *p* = 0.031). The LW-LC N400 difference waves were also more negative than those elicited by HW-HC social groups (M = −2.42 μV; *p* = 0.363) and LW-HC social groups (M = −2.08 μV; *p* = 0.233), but differences did not reach significance. No other comparisons between social groups approached significance (*p*s > 0.775). 

***LPC*.** Similar to the statistical analyses of the P2 and N400 component, analyses of the LPC were conducted on mean ERP amplitudes of the given ROI (F1, Fz, F2, FC1, FCz, FC2, C1, Cz, C2, CP1, CPz, CP2) in the 500-700 ms time-window. The analysis of LPC amplitudes revealed a significant main effect of group [F (3, 54) = 8.34, *p* < 0.001, partial *η*^2^ = 0.32] and congruency [F (1, 18) = 19.24, *p* < 0.001, partial *η*^2^ = 0.52]. Moreover, the interaction between group and congruency was significant as well, F (3, 54) = 2.88, *p* = 0.044, partial *η*^2^ = 0.14.

**Figure 5:**
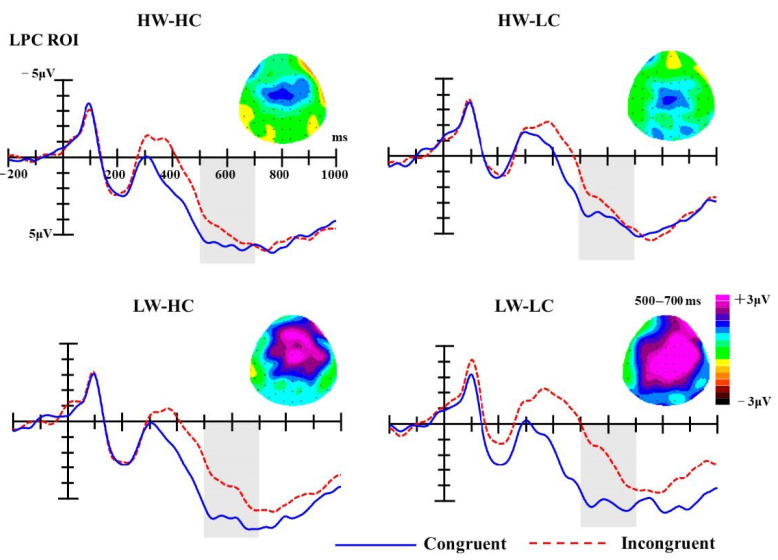
The grand-average ERPs at selected ROI electrodes (F1, Fz, F2, FC1, FCz, FC2, C1, Cz, C2, CP1, CPz, CP2); the shaded areas mark LPC components (500–700 ms). Topographies of late positive component (LPC) difference waves (incongruent minus congruent trials) for four SCM quadrants.

As presented in Figure 5, simple effect analyses for the interaction showed that LPC amplitudes elicited by congruent targets were significantly more positive than those elicited by incongruent targets for the LW-LC groups (M _congruent_ = 5.32 μV, M _incongruent_ = 2.45 μV, MD _congruent__-incongruent_ = 2.87 μV, SE = 0.59, *p* < 0.001) and LW-HC groups (M _congruent_ = 6.45 μV, M _incongruent_ = 4.51 μV, MD _congruent__-incongruent_ = 2.04 μV, SE = 0.65, *p* = 0.006). While the LPC amplitudes elicited by congruent targets were also more positive than those elicited by incongruent targets for the HW-HC (M _congruent_ = 5.73 μV, M _incongruent_ = 4.92 μV, MD _congruent-incongruent_ = 0.81 μV, SE = 0.63, *p* = 0.216) and HW-LC (M _congruent_ = 4.14 μV, M _incongruent_ = 3.37 μV, MD _congruent-incongruent_ = 0.77 μV, SE = 0.69, *p* = 0.280) social groups, these differences failed to reach significant. 

### 3.3. Discussion

Predictions for Study 2 were largely supported. Participants responded more quickly on stereotype congruent trials than incongruent trials for HW-HC, HW-LC, and LW-HC social groups. Replicating the results from Study 1, an opposite RT pattern was observed for LW-LC groups. Specifically, participants responded more quickly on incongruent than congruent trials for these groups. This highlights that LW-LC groups are perceived and processed differently than social groups from the other three SCM clusters. Results from the ERP data further support this conclusion. Replicating previous research [39,40,41,42,67,83], stereotype incongruent trials elicited more negative N400 amplitudes than congruent trials for all SCM quadrants. Extending this research and confirming predictions, the N400 effect was most pronounced for the LW-LC groups. Additionally, larger P2 amplitudes were elicited by stereotype congruent trials than incongruent trials, but only for the LW-LC groups, and LPC amplitudes were larger on congruent trials than incongruent trials, but only for the LW-LC and LW-HC groups.

## 4. General Discussion

The present research had two primary goals: (1) expand the investigation of stereotype activation/priming to social groups that represent all clusters under the framework of SCM, and (2) use event-related potentials (ERPs) to elucidate the timing and nature of stereotypical processing for these groups. A sequential priming paradigm was used to investigate stereotype activation in response times (Studies 1 and 2) and ERPs (Study 2) for a variety of social groups under the framework of SCM. Across both studies, response times revealed typical stereotype priming effects (faster RTs on stereotype congruent than incongruent trials) for HW-HC, HW-LC, and LW-HC social groups while a reverse priming pattern (slower RTs on congruent than incongruent trials) was observed for LW-LC social groups. ERP results from Study 2 further highlighted the unique processing of LW-LC groups. Amplitudes were larger on stereotype congruent than incongruent trials for P2 components, but only for the LW-LC groups. N400 amplitudes were larger on stereotype incongruent than congruent trials for all four SCM quadrants, while this difference on N400 was largest for LW-LC groups. Finally, LPC amplitudes were larger on stereotype congruent than incongruent trials, but only for the low warmth (LW-LC and LW-HC) groups. These results reveal a great deal about the way various SCM groups are processed, and provide further evidence that LW-LC quadrant are processed differently than other three SCM quadrants. 

The unique status of LW-LC groups is evident very early in the stream of cognitive processing. P2 amplitudes elicited by stereotype congruent trials were greater than those elicited by stereotype incongruent trials, but only on trials that used a LW-LC group as the prime. Previous research demonstrates that larger P2 amplitudes are associated with perceptions of threatening and/or negative stimuli [78,98,99]. The heightened P2 amplitudes elicited on congruent trials for LW-LC groups thus indicates that these groups attract greater attention because they are perceived as more negative and/or threatening than other groups.

Additional evidence that LW-LC groups are processed uniquely was observed further down in the cognitive stream, in both N400 and LPC amplitudes. The N400 component is thought to reflect the dynamic activation of associations in long-term memory, in the sense that N400 amplitudes elicited by a given stimulus will be smaller when the associations typically activated by that stimulus are already partially or fully activated by other stimuli or experiences [81]. Applied to the current studies, exposure to a social category (prime stimulus) activates information about this group in long-term memory. When the category is followed by a stereotype congruent target, this pre-activation of information facilitates processing of the target stimulus, resulting in a smaller N400 amplitude than if the target stimulus is stereotypically incongruent with the primed social group. This was the case for all SCM groups in the present research, but the effect was particularly pronounced for the LW-LC groups. More specifically, the amplitudes of N400 difference waves for LW-LC groups are larger than those for the other SCM groups. This suggests that the semantic integration for LW-LC groups is harder than for the other groups. As a whole, these results replicate previous N400 stereotype research [39,40,41,67,83] and extend it in two important ways—(1) they demonstrate that the N400 can be used to examine stereotypes for a wide range of social groups under the framework of SCM, and (2) they add to existing evidence for the unique status of LW-LC groups. 

While the N400 results were expected, it was somewhat surprising that congruency also modulated LPC amplitudes for LW-LC and LW-HC groups. Specifically, congruent trials elicited larger LPC amplitudes than incongruent trials for low warmth (LW) groups. A potential explanation for these results comes from research that has connected the LPC to avoidance tendencies. In previous research, the LPC has been linked to the regulation of approach-avoidance tendencies, with larger amplitudes accompanying avoidance tendencies than approach tendencies [100]. Heightened LPC amplitudes on congruent trials for LW-LC and LW-HC groups may therefore reflect a general tendency to avoid LW groups. This aligns with previous research where approach–avoidance tendencies have been connected to warmth judgments (e.g., [101]). It also makes sense within the theoretical framework of the SCM. According to the SCM, the warmth dimension is prioritized over the competence dimension (receiving both earlier attention and greater weight) due to the evolutionary importance of discerning good or ill intentions in others [20]. This could explain why congruency modulated the LPC for both LW-LC and LW-HC groups—both groups are perceived primarily in terms of their warmth, and avoidance tendencies are correspondingly activated. The activation of avoidance tendencies might also explain the behavioral results observed in the present research. 

The stream of unique processing for LW-LC groups culminate in a very distinct pattern for response times (RTs). Specifically, we observed typical RT priming effects for HW-HC, HW-LC, and LW-HC groups, but an opposite pattern (longer RTs on congruent trials than incongruent trials) for LW-LC groups. This reverse priming effect for LW-LC groups has been replicated in two additional experiments in Yang et al. [87], and reverse priming effects, in general, have been observed in a variety of conditions in past research. For example, reverse priming effects have been reported when a long SOA is used, when each trial contains two primes, when instructions emphasize accuracy over speed, and when primes are extreme (for review, see [102]). Of these conditions, only the condition employing extreme primes applies to the present research. Specifically, the LW-LC groups may be perceived as extreme with regard to their low warmth and low competence, relative to the other groups used as primes. Their extremity would cause these groups to stand out and subtly alert participants to their potentially biasing influence on responses [103]. This may, in turn, activate an unconscious goal to correct for this bias [103] (for review, see [104]), resulting in slowed responses on congruent trials. Considering the difference on P2 found on LW-LC groups, P2 might also be an indicator of unconscious activation of egalitarian goal or unconscious activation of motivation to correct bias [104,105,106,107]. However, this possibility needs further empirical support.

Alternatively, the reverse priming effect may reflect an avoidance behavioral tendency toward these groups, particularly since these groups elicit the emotion of disgust [12]. Previous research using disgust facial cues suggests that disgust strongly elicits avoidance tendencies, which ultimately diverts, rather than attracts, attention to stimuli [108,109]. In this line of research, dot-probe judgments were faster on validly cued trials than invalidly cued trials when fearful or angry facial cues were used. However, when disgust facial cues were used, judgments were slower on validly cued trials. In other words, the RT pattern was reversed on disgust facial cue trials [108,109]. Applied to the present research, LW-LC congruent trials might elicit disgust and attendant avoidance tendencies, significantly slowing responses on congruent trials relative to incongruent trials. Because disgust is not the dominant emotion elicited by other SCM groups [12], it would not interfere with responses on congruent trials for these groups. If the activation of disgust and avoidance explains the reversed RT pattern, we would see a significant slow-down on congruent trials for LW-LC groups, but much less or no difference in RTs between LW-LC and other SCM groups for incongruent trials. The difference in RTs between LW-LC and other SCM groups for incongruent trials was non-significant (Study 1) or relatively small (Study 2), meaning that participants responded to incongruent trials fairly similarly for all SCM groups. However, RTs were significantly longer for LW-LC groups for congruent trials in both studies, meaning that participants responded much more slowly on congruent trials for LW-LC groups than for the other SCM groups.

One final possible explanation for the reverse priming effect involves distrust. According to the SCM and BIAS map, LW-LC groups can easily become a target of distrust [31,110,111], and previous research has found that distrust can facilitate processing of inconsistent information while inhibiting the processing of congruent information (e.g., [112]; for review, see [113]). Additionally, other research has found that distrust primarily affects stereotyping by reducing the application of stereotypes, as opposed to reducing the activation of stereotypes [114]. This could explain why a reverse-priming effect was observed in response times, but the anticipated pattern was observed for LW-LC groups for the N400 component. All these potential explanations, however, are speculative and require direct testing in future studies. 

Taken as a whole, the results from the present research suggest that LW-LC groups may attract additional attention early in processing (as evidenced in P2 activity), but ultimately attention is repelled away from them as processing culminates in avoidance behavioral tendencies. Although share some common neural underpinnings (temporoparietal junction and right anterior temporal lobe [31]), results from the present research also highlight some important differences between LW-LC groups and LW-HC groups. LW-LC groups were immediately perceived as more negative or threatening (P2), activated avoidance predispositions (LPC), and ultimate elicited avoidance behavioral responses (RTs). LW-HC groups also appeared to activate avoidance tendencies (LPC), but they did not attract early attention, were not immediately perceived negatively, and did not elicit avoidance behavioral responses. According to the BIAS map [12], LW-LC groups elicit the emotion of disgust and both active and passive forms of harmful behavior (directed toward members of these groups). The BIAS map does not identify any general conditions when these groups would elicit facilitating (helpful/friendly) behavior from others. These groups may therefore be automatically and unconditionally perceived negatively, activating avoidance predispositions that are translated into action. This would explain their differential processing from perception to behavior in the present research. 

Because of their mixed stereotype content (low warmth but high competence), LW-HC groups elicit the more ambiguous emotion of envy and either passive facilitation behavior or active harm behaviors. The BIAS map proposes that the type of behavior LW-HC groups elicit (facilitation or harm) depends on the situation and circumstances. In times of stability and success, people will passively facilitate (cooperate) with these groups because their high status makes it advantageous to do so. In times of instability and misfortune, however, people can turn on these groups as scapegoats and may actively try to harm them [12]. LW-HC groups would therefore not be automatically and unconditionally perceived negatively, hence the absence of differential processing of these groups in P2. Due to the primacy of the warmth dimension, it is reasonable to assume that LW-HC groups would elicit avoidance tendency, hence differential processing in the LPC occurred. However, absent any clear signals of instability or misfortune, it would be more advantageous to cooperate with, rather than avoid, these groups in LW-HC quadrant. Thus, these avoidance tendencies were not translated into avoidance behavioral tendencies in the present research. It is possible that an opposite RT pattern would be observed for these groups if signals of instability/misfortune were introduced into the experimental context. This is an interesting question to pursue in future research. 

Some limitations and future directions should be mentioned in the current study. First of all, researchers should seek to replicate these results in other populations. Both of the current samples were composed of Chinese undergraduate students. In one sense, this helps improve the overall diversity of samples in psychological research, which tends to skew heavily toward Western cultures. On the other hand, the results may apply specifically to Chinese individuals. We consider this unlikely, though, given that the basic response time and N400 effects observed in the present research replicate past research conducted with Western samples. Even so, replications with other populations and non-student samples would increase confidence in the generalizability of the results. Moreover, the current findings were obtained at the intergroup level. Considered that SCM was also applied in interpersonal perception [21,22,23], it would be interesting to explore the reverse priming effect and its mechanism at the inter-individual level. Recently, a framework [115] was proposed to integrate five different but connected models (including SCM [11], dimensional compensation model [116], dual perspective model [117], behavioral regulation model [118] and agency beliefs-communion model [119]). Four types of moderators (type of target, number of targets, perceiver-target relationship and context) were raised to explain contradicts among these models. The present results were obtained only under the framework of SCM, whether these results could be replicated under the newly integrated framework or other four models still remains unknown and the potential moderating role of these four kinds of moderators on reverse priming effect and traditional priming effect would be a fruitful field in the future. Lastly, the perceived warmth and competence for all four SCM quadrants fit SCM map well when checked individually, however, when validating the groups across SCM categories, the competence dimension did not fit perfectly while warmth dimension was balanced among four SCM quadrants in the current study. Specifically, the difference on competence ratings between LW-HC and HW-LC did not reach significant (*p* = 0.225) and HW-LC was perceived more competent than LW-LC (*p* = 0.006), even though they are both labeled as LC quadrants. Moreover, the SCM quadrants identified from pretest were the results of relative placement rather than the ideal absolute placement (see [88] for details). This limitation would be a potential confound and need addressed in future studies.

## 5. Conclusions

The present research makes several meaningful contributions to the literature. First, as far as we know, this is the one of the first studies to investigate the neural mechanism underlying reverse priming effect. Secondly, it verifies that the N400 ERP component can be used as a measure of stereotype activation for groups within all quadrants of the SCM. Thirdly, it adds to previous research evidence that suggests LW-LC social groups are processed differently than other SCM groups, and extends this research by elucidating the various time points when these groups are uniquely processed. Finally, it highlights the utility of ERPs for differentiating between the various SCM groups with regard to the sequence and timing of their processing. When combined with fMRI data, ERP data can provide a more complete and thorough understanding of the neural processes that give rise to biased/stereotypical processing of, as well as potentially discriminatory behaviors toward, these groups.

The present results demonstrate that the reverse priming effect is robust and exist among various LW-LC social groups. The underlying mechanism might involve early attention attraction (P2), followed by difficulty on semantic integration (N400) and avoidance behavioral tendency (LPC). Future studies are urgently needed to elucidate the moderators of the reverse priming effect and the mechanism underlying it.

## Appendix A

**Table A1:** The prime words and target words for four SCM quadrants.

SCM Quadrants	Prime Words in Chinese	Prime Words in English	Stereotype Congruent Target Words in Chinese	Stereotype Congruent Target Words in English	Stereotype Incongruent Target Words in Chinese	Stereotype Incongruent Target Words in English
**HW-HC**	军人	soldiers	保家卫国	protect our homes and defend our country	重利益	regard interests as highly important
			威严	dignified	欺软怕硬	bully the weak and fear the strong
			坚毅	fortitude	瘦弱	thin and weak
			正义	righteous	可恶	hateful
			正直	upstanding	爱唠叨	nagging
	消防员	firemen	工作危险	dangerous work	喊口号	shout slogans
			行动迅速	act quickly	行动缓慢	slow movement
			灭火救人	put out the fire and rescue people	不办实事	don’t get things done
			令人尊敬	respectable	唯利是图	seek nothing but profits
			敬业	dedicated	凶残	merciless
			勇敢	brave	无责任心	without the sense of responsibility
	大学教师	professors	有学问	scholarly	狡猾	crafty
			知识渊博	knowledgeable	凶残	cruel
			教书育人	imparting knowledge and educating people	游手好闲	idle
	心理咨询师	psychotherapists	善于观察	good at observing	没人性	inhuman
			懂人心	empathetic	缺少关爱	lack of love and care
			有耐心	have much patience	极端	go to extremes
			温和	gentle	没有同情心	without compassion
			帮助他人	helping others	凶狠	fierce
	空姐	air hostesses	礼仪好	good manners	威严	dignified
			微笑服务	service with a smile	残暴	brute
			有气质	graceful	颓废	decadence
			漂亮	pretty	白发苍苍	grey-haired
			美丽大方	beautiful and gracious	心理扭曲	psychological distortion
			举止优雅	elegant manner	蛮横	rude and unreasonable
	瑜伽教练	yoga instructors	柔韧性强	great flexibility	精神涣散	spiritless
			体形优美	beautiful shape	大腹便便	big-bellied
			身材好	good figure	没有上进心	no desire to advance
			健美	well-built	邋遢	slovenly
			健康	athletic	脏	dirty
**HW-LC**	老年人	elderly	需要照顾	need to be looked after	体形优美	beautiful shape
			需要关爱	need love and care	柔韧性强	great flexibility
			爱唠叨	nagging	身材好	good figure
			白发苍苍	grey-haired	残暴	brutal
			行动缓慢	slow movement	行动迅速	act quickly
			慈祥	kindly	挥霍	spend extravagantly
	农民	farmers	辛苦	laborious work	知识渊博	knowledgeable
			种田	farming	私生活混乱	promiscuous
			勤劳	industrious	奢侈	spend extravagantly
			憨厚老实	simple and honest	懒惰	lazy
			收入低	low-income	官腔官调	bureaucratic
	家庭主妇	housewives	做饭带孩子	cooking and taking care of children	留学	study abroad
			顾家	value family	保家卫国	protect our homes and defend our country
			做家务	keep house	贪污腐败	corrupt
			贤惠	virtuous	傲慢	arrogant
			勤劳	industrious	形式主义	formalism
	农民工	migrant workers	辛苦	laborious work	有知识	educated
			劳累	overworked	工作清闲	work at leisure
			生活艰辛	life is tough	工作稳定	stable job
			弱势群体	vulnerable group	官腔	official jargon
	留守儿童	left-behind children	渴望陪伴	eager to be accompanied by parents	见识广	well-informed
			缺少关爱	lack of love and care	有钱	wealthy
			孤独	lonely	美丽大方	beautiful and gracious
			可怜	pitiful	外语好	good foreign language
	清洁工	cleaning workers	辛苦	laborious work	有气质	graceful
			早起	early rising	有学问	scholarly
			朴实	simple	收入高	high income
			可敬	worthy of respect	铁饭碗	has a stable lifelong job
			勤劳	industrious	漂亮	pretty
			工资低	low salary	待遇好	well-paid
**LW-HC**	商人	businessmen	重利益	regard interests as highly important	渴望陪伴	eager to accompany
			有钱	wealthy	贫穷	poor
			精明	shrewd	憨厚老实	simple and honest
			唯利是图	seek nothing but profits	做家务	keep house
	海归	overseas returnees	留学	study abroad	监狱	prison
			外语好	good foreign language	萎靡	flattened
			见识广	well-informed	种田	farming
			有知识	educated	工资低	low salary
	公务员	government workers	工作稳定	stable job	绯闻多	need more concern
			铁饭碗	has a stable lifelong job	需要照顾	need to be looked after
			工作清闲	work at leisure	堕落	decadent
			待遇好	well-paid	工作危险	dangerous work
	政府官员	government officials	官腔	official jargon	有耐心	have much patience
			形式主义	formalism	敬业	dedicated
			官腔官调	bureaucratic	孤独	lonely
			喊口号	shout slogans	弱势群体	vulnerable group
			不办实事	don’t get things done	朴实	simple
			贪污腐败	corrupt	做饭带孩子	cooking and taking care of children
			大腹便便	big-bellied	骨瘦如柴	skinny
	演艺明星	stars in showbiz	收入高	high income	无所事事	slothful
			绯闻多	more scandal	啃老	depend on parents for living
			光鲜亮丽	glamorous	衣衫褴褛	shabbily dressed
			漂亮	pretty	收入低	low-income
			私生活混乱	promiscuous	灭火救人	put out the fire and rescue people
			好看	good-looking	形容枯槁	haggard
	富人	rich	有钱	wealthy	生活艰辛	life is tough
			奢侈	extravagant	需要关爱	need love and care
			傲慢	arrogant	劳累	overworked
			狡猾	crafty	可怜	pitiful
			挥霍	spend extravagantly	辛苦	laborious work
**LW-LC**	罪犯	criminals	监狱	prison	慈祥	kindly
			可恶	hateful	令人尊敬	respectable
			凶狠	fierce	正义	righteous
			残暴	violent and cruel	教书育人	imparting knowledge and educating people
	无业游民	unemployed	游手好闲	idle	辛苦	laborious work
			无所事事	slothful	早起	early bird
			无责任心	irresponsible	好看	good-looking
			啃老	depend on parents for living	勤劳	industrious
			没有上进心	no desire to advance	勇敢	courageous
	乞丐	beggars	脏	dirty	健美	well-built
			衣衫褴褛	shabbily dressed	光鲜亮丽	glamorous
			邋遢	slovenly	有钱	wealthy
			懒惰	lazy	漂亮	pretty
			贫穷	poor	勤劳	industrious
	吸毒者	drug addicts	颓废	decadence	健康	fitness
			堕落	decadent	辛苦	laborious work
			萎靡	flattened	勤劳	industrious
			精神涣散	spiritless	善于观察	good at observing
			骨瘦如柴	skinny	坚毅	fortitude
			形容枯槁	haggard	精明	shrewd
			瘦弱	thin and weak	顾家	value family
	恐怖分子	terrorists	极端	go to extremes	帮助他人	helping others
			残暴	brutal	微笑服务	service with a smile
			凶残	cruel	正直	upstanding
			心理扭曲	psychological distortion	举止优雅	elegant manner
			没人性	inhuman	礼仪好	good manners
	城管	urban management officers	欺软怕硬	bully the weak and fear the strong	温和	gentle
			蛮横	rude and unreasonable	懂人心	empathetic
			凶残	merciless	贤惠	virtuous
			没有同情心	without compassion	可敬	worthy of respect

## References

[1] Macrae C.N., Bodenhausen G.V. (2000). Social cognition: Thinking categorically about others. Annu. Rev. Psychol..

[2] Ellemers N. (2018). Gender stereotypes. Annu. Rev. Psychol..

[3] Blair I.V., Banaji M.R. (1996). Automatic and controlled processes in stereotype priming. J. Personal. Soc. Psychol..

[4] Devine P.G. (1989). Stereotypes and prejudice: Their automatic and controlled components. J. Personal. Soc. Psychol..

[5] Macrae C.N., Milne A.B., Bodenhausen G.V. (1994). Stereotypes as energy-saving devices: A peek inside the cognitive toolbox. J. Personal. Soc. Psychol..

[6] Oakhill J., Garnham A., Reynolds D. (2005). Immediate activation of stereotypical gender information. Mem. Cognit..

[7] Amodio D.M., Cikara M. (2021). The Social Neuroscience of Prejudice. Annu. Rev. Psychol..

[8] Quadflieg S., Macrae C.N. (2011). Stereotypes and stereotyping: What’s the brain got to do with it?. Eur. Rev. Soc. Psychol..

[9] Amodio D.M. (2014). The neuroscience of prejudice and stereotyping. Nat. Rev. Neurosci..

[10] Blair I.V. (2002). The Malleability of Automatic Stereotypes and Prejudice. Personal. Soc. Psychol. Rev..

[11] Fiske S.T., Cuddy A.J.C., Glick P., Xu J. (2002). A model of (often mixed) stereotype content: Competence and warmth respectively follow from perceived status and competition. J. Personal. Soc. Psychol..

[12] Cuddy A.J.C., Fiske S.T., Glick P. (2007). The BIAS map: Behaviors from intergroup affect and stereotypes. J. Personal. Soc. Psychol..

[13] Fiske S.T., Cuddy A.J.C., Glick P. (2007). Universal dimensions of social cognition: Warmth and competence. Trends Cogn. Sci..

[14] Fiske S.T. (2015). Intergroup biases: A focus on stereotype content. Curr. Opin. Behav. Sci..

[15] Fiske S.T. (2018). Stereotype content: Warmth and competence endure. Curr. Dir. Psychol. Sci..

[16] Cuddy A.J.C., Fiske S.T., Kwan V.S.Y., Glick P., Demoulin S., Leyens J.P., Bond M.H., Croizet J.C., Ellemers N., Sleebos E. (2009). Stereotype content model across cultures: Towards universal similarities and some differences. Br. J. Soc. Psychol..

[17] Wu S.J., Bai X., Fiske S.T. (2018). Admired rich or resented rich? How two cultures vary in envy. J. Cross Cultur. Psychol..

[18] Bye H.H., Herrebrøden H., Hjetland G.J., Røyset G.Ø., Westby L.L. (2014). Stereotypes of Norwegian social groups. Scand. J. Psychol..

[19] Grigoryev D., Fiske S.T., Batkhina A. (2019). Mapping ethnic stereotypes and their antecedents in Russia: The stereotype content model. Front. Psychol..

[20] Cuddy A.J.C., Fiske S.T., Glick P. (2008). Warmth and competence as universal dimensions of social perception: The stereotype content model and the BIAS map. Adv. Exp. Soc. Psychol..

[21] Russell A.M.T., Fiske S.T. (2008). It’s all relative: Competition and status drive interpersonal perception. Eur. J. Soc. Psychol..

[22] Simon J.C., Styczynski N., Gutsell J.N. (2020). Social perceptions of warmth and competence influence behavioral intentions and neural processing. Cogn. Affect. Behav. Neurosci..

[23] Abele A.E., Yzerbyt V. (2020). Body posture and interpersonal perception in a dyadic interaction: A Big Two Analysis. Eur. J. Soc. Psychol..

[24] Harris L.T., Fiske S.T. (2006). Dehumanizing the lowest of the low neuroimaging responses to extreme out-groups. Psychol. Sci..

[25] Harris L.T., Fiske S.T. (2007). Social groups that elicit disgust are differentially processed in mPFC. Soc. Cogn. Affect. Neurosci..

[26] Harris L.T., Fiske S.T. (2009). Social neuroscience evidence for dehumanised perception. Eur. Rev. Soc. Psychol..

[27] Haslam N., Loughnan S. (2014). Dehumanization and infrahumanization. Annu. Rev. Psychol..

[28] Krendl A.C. (2016). An fMRI investigation of the effects of culture on evaluations of stigmatized individuals. NeuroImage.

[29] Cikara M., Farnsworth R.A., Harris L.T., Fiske S.T. (2010). On the wrong side of the trolley track: Neural correlates of relative social valuation. Soc. Cogn. Affect. Neurosci..

[30] Dricu M., Bührer S., Hesse F., Eder C., Posada A., Aue T. (2018). Warmth and competence predict overoptimistic beliefs for out-group but not in-group members. PLoS ONE.

[31] Dricu M., Schüpbach L., Bristle M., Wiest R., Moser D.A., Aue T. (2020). Group membership dictates the neural correlates of social optimism biases. Sci. Rep..

[32] Moser D.A., Dricu M., Wiest R., Schüpbach L., Aue T. (2020). Social optimism biases are associated with cortical thickness. Soc. Cogn. Affect. Neurosci..

[33] Kawakami K., Young H., Dovidio J.F. (2002). Automatic stereotyping: Category, trait, and behavioral activations. Personal. Soc. Psychol. Bull..

[34] Macrae C.N., Bodenhausen G.V., Milne A.B. (1995). The dissection of selection in person perception: Inhibitory processes in social stereotyping. J. Personal. Soc. Psychol..

[35] Pendry L.F., Macrae C.N. (1996). What the disinterested perceiver overlooks: Goal-directed social categorization. Personal. Soc. Psychol. Bull..

[36] Sinclair L., Kunda Z. (1999). Reactions to a black professional: Motivated inhibition and activation of conflicting stereotypes. J. Personal. Soc. Psychol..

[37] Kidder C.K., White K.R., Hinojos M.R., Sandoval M., Crites S.L. (2018). Sequential stereotype priming: A meta-analysis. Personal. Soc. Psychol. Rev..

[38] White K.R., Danek R.H., Herring D.R., Taylor J.H., Crites S.L. (2018). Taking priming to task: Variations in stereotype priming effects across participant task. Soc. Psychol..

[39] Wang P., Yang Y., Zhao L. (2010). The activation of stereotypes: Behavioral and ERPs evidence. Acta Psychol. Sin..

[40] White K.R., Crites S.L., Taylor J.H., Corral G. (2009). Wait, what? Assessing stereotype incongruities using the N400 ERP component. Soc. Cogn. Affect. Neurosci..

[41] Wang P., Yang Y., Tan C., Chen Q., Van Cantfort T.E. (2017). Gender stereotype activation versus lexical semantic activation: An ERP study. J. Gen. Psychol..

[42] Yang Y., Wang P., Yin Z., Chen Q., Feng X. (2015). The pattern and neural correlates of unintentional stereotype activation. Acta Psychol. Sin..

[43] Zhang X., Li Q., Sun S., Zuo B. (2018). The time course from gender categorization to gender stereotype activation. Soc. Neurosci..

[44] Plaza M., Boiché J., Brunel L., Ruchaud F. (2017). Sport = male but not all sports: Investigating the gender stereotypes of sport activities at the explicit and implicit levels. Sex Roles.

[45] Tsamadi D., Falbén J.K., Persson L.M., Golubickis M., Caughey S., Sahin B., Macrae C.N. (2020). Stereotype-based priming without stereotype activation: A tale of two priming tasks. Q. J. Exp. Psychol..

[46] Müller F., Rothermund K. (2014). What does it take to activate stereotypes? Simple primes don’t seem enough: A replication of stereotype activation (Banaji & Hardin, 1996; Blair & Banaji, 1996). Soc. Psychol..

[47] Banaji M.R., Hardin C.D. (1996). Automatic stereotyping. Psychol. Sci..

[48] Kimura A., Wada Y., Goto S., Tsuzuki D., Cai D., Oka T., Dan I. (2009). Implicit gender-based food stereotypes. Semantic priming experiments on young Japanese. Appetite.

[49] Macrae C.N., Cloutier J. (2009). A matter of design: Priming context and person perception. J. Exp. Soc. Psychol..

[50] Macrae C.N., Martin D. (2007). A boy primed Sue: Feature-based processing and person construal. Eur. J. Soc. Psychol..

[51] Macrae C.N., Mitchell J.P., Pendry L.F. (2002). What’s in a forename? Cue familiarity and stereotypical thinking. J. Exp. Soc. Psychol..

[52] Gawronski B., Deutsch R., Mbirkou S., Seibt B., Strack F. (2008). When “just say no” is not enough: Affirmation versus negation training and the reduction of automatic stereotype activation. J. Exp. Soc. Psychol..

[53] Kawakami K., Dovidio J.F. (2001). The reliability of implicit stereotyping. Personal. Soc. Psychol. Bull..

[54] Todd A.R., Thiem K.C., Neel R. (2016). Does seeing faces of young black boys facilitate the identification of threatening stimuli?. Psychol. Sci..

[55] Ito T.A., Tomelleri S. (2017). Seeing is not stereotyping: The functional independence of categorization and stereotype activation. Soc. Cogn. Affect. Neurosci..

[56] Wittenbrink B., Judd C.M., Park B. (1997). Evidence for racial prejudice at the implicit level and its relationship with questionnaire measures. J. Personal. Soc. Psychol..

[57] Wittenbrink B., Judd C.M., Park B. (2001). Evaluative versus conceptual judgments in automatic stereotyping and prejudice. J. Exp. Soc. Psychol..

[58] Amodio D.M., Harmon-Jones E., Devine P.G., Curtin J.J., Hartley S.L., Covert A.E. (2004). Neural signals for the detection of unintentional race bias. Psychol. Sci..

[59] Dickter C.L., Kittel J.A. (2012). The effect of stereotypical primes on the neural processing of racially ambiguous faces. Soc. Neurosci..

[60] Govorun O., Payne B.K. (2006). Ego—Depletion and prejudice: Separating automatic and controlled components. Soc. Cogn..

[61] Jones C.R., Fazio R.H. (2010). Person categorization and automatic racial stereotyping effects on weapon identification. Personal. Soc. Psychol. Bull..

[62] Kawakami K., Dion K.L., Dovidio J.F. (1999). Implicit stereotyping and prejudice and the primed Stroop task. Swiss J. Psychol..

[63] Kubota J.T., Ito T.A. (2014). The role of expression and race in weapons identification. Emotion.

[64] Payne B.K. (2001). Prejudice and perception: The role of automatic and controlled processes in misperceiving a weapon. J. Personal. Soc. Psychol..

[65] Payne B.K. (2005). Conceptualizing control in social cognition: How executive functioning modulates the expression of automatic stereotyping. J. Personal. Soc. Psychol..

[66] Stewart B.D., Payne B.K. (2008). Bringing automatic stereotyping under control: Implementation intentions as efficient means of thought control. Personal. Soc. Psychol. Bull..

[67] Hehman E., Volpert H.I., Simons R.F. (2014). The N400 as an index of racial stereotype accessibility. Soc. Cogn. Affect. Neurosci..

[68] Freeman J.B., Ambady N. (2011). A dynamic interactive theory of person construal. Psychol. Rev..

[69] Kawakami K., Amodio D.M., Hugenberg K. (2017). Intergroup perception and cognition: An integrative framework for understanding the causes and consequences of social categorization. Advances in Experimental Social Psychology.

[70] Krieglmeyer R., Sherman J.W. (2012). Disentangling stereotype activation and stereotype application in the stereotype misperception task. J. Personal. Soc. Psychol..

[71] Jia L., Dickter C.L., Luo J., Xiao X., Yang Q., Lei M., Qiu J., Zhang Q. (2012). Different brain mechanisms between stereotype activation and application: Evidence from an ERP study. Int. J. Psychol..

[72] Amodio D.M., Bartholow B.D., Ito T.A. (2014). Tracking the dynamics of the social brain: ERP approaches for social cognitive and affective neuroscience. Soc. Cogn. Affect. Neurosci..

[73] Ito T.A., Kubota J.T. (2019). Bioelectrical echoes from a career at the cutting edge: John Cacioppo’s legacy and the use of ERPs in social psychology. Soc. Neurosci..

[74] Wang P., Yang Y., Tan C., Zhao X., Liu Y., Lin C. (2016). Stereotype activation is unintentional: Behavioural and event-related potenials evidence. Int. J. Psychol..

[75] Pesciarelli F., Scorolli C., Cacciari C. (2019). Neural correlates of the implicit processing of grammatical and stereotypical gender violations: A masked and unmasked priming study. Biol. Psychol..

[76] Luck S.J., Hillyard S.A. (1994). Electrophysiological correlates of feature analysis during visual search. Psychophysiology.

[77] Dickter C.L., Bartholow B.D. (2007). Racial ingroup and outgroup attention biases revealed by event-related brain potentials. Soc. Cogn. Affect. Neurosci..

[78] Ito T.A., Urland G.R. (2003). Race and gender on the brain: Electrocortical measures of attention to the race and gender of multiply categorizable individuals. J. Personal. Soc. Psychol..

[79] Ito T.A., Urland G.R. (2005). The influence of processing objectives on the perception of faces: An ERP study of race and gender perception. Cogn. Affect. Behav. Neurosci..

[80] Willadsen-Jensen E.C., Ito T.A. (2006). Ambiguity and the timecourse of racial perception. Soc. Cogn..

[81] Kutas M., Federmeier K.D. (2011). Thirty years and counting: Finding meaning in the N400 component of the event related brain potential (ERP). Annu. Rev. Psychol..

[82] Bentin S., McCarthy G., Wood C.C. (1985). Event-related potentials, lexical decision and semantic priming. Electroencephalogr. Clin. Neurophysiol..

[83] Wang L., Ma Q., Song Z., Shi Y., Wang Y., Pfotenhauer L. (2011). N400 and the activation of prejudice against rural migrant workers in China. Brain Res..

[84] Osterhout L., Bersick M., McLaughlin J. (1997). Brain potentials reflect violations of gender stereotypes. Mem. Cognit..

[85] Hammer A., Jansma B.M., Lamers M., Münte T.F. (2005). Pronominal reference in sentences about persons or things: An electrophysiological approach. J. Cogn. Neurosci..

[86] Xu X., Jiang X., Zhou X. (2013). Processing biological gender and number information during Chinese pronoun resolution: ERP evidence for functional differentiation. Brain Cogn..

[87] Yang Y., Xu Q., Zhu T., Zheng X., Dong X., Chen Q. (2019). The behavioral patterns of stereotype activation among four different warmth-competence social groups based on Stereotype Content Model. Acta Psychol. Sin..

[88] Ji Z., Chen Q., Fan X., Xu Q., Yang Y. (2020). Stereotypes of Social Groups in Mainland China in Terms of Warmth and Competence: Evidence from a Large Undergraduate Sample. https://psyarxiv.com/dbh3v/.

[89] Katz D., Braly K. (1933). Racial stereotypes of one hundred college students. J. Abnorm. Soc. Psychol..

[90] Ratcliff R. (1993). Methods for dealing with reaction time outliers. Psychol. Bull..

[91] Bartholow B.D. (2010). On the role of conflict and control in social cognition: Event-related brain potential investigations. Psychophysiology.

[92] Wentura D., Degner J. (2010). A practical guide to sequential priming and related tasks. Handbook of Implicit Social Cognition: Measurement, Theory, and Applications.

[93] Conrey F.R., Sherman J.W., Gawronski B., Hugenberg K., Groom C.J. (2005). Separating multiple processes in implicit social cognition: The quad model of implicit task performance. J. Personal. Soc. Psychol..

[94] Hütter M., Klauer K.C. (2016). Applying processing trees in social psychology. Eur. Rev. Soc. Psychol..

[95] Johnson D.J., Hopwood C.J., Cesario J., Pleskac T.J. (2017). Advancing research on cognitive processes in social and personality psychology: A hierarchical drift diffusion model primer. Soc. Psychol. Personal. Sci..

[96] Gratton G., Coles M.G., Donchin E. (1983). A new method for off-line removal of ocular artifact. Electroencephalogr. Clin. Neurophysiol..

[97] Keil A., Debener S., Gratton G., Junghöfer M., Kappenman E.S., Luck S.J., Luu P., Miller G.A., Yee C.M. (2014). Committee report: Publication guidelines and recommendations for studies using electroencephalography and magnetoencephalography. Psychophysiology.

[98] Carretié L., Martín-Loeches M., Hinojosa J.A., Mercado F. (2001). Emotion and attention interaction studied through event-related potentials. J. Cogn. Neurosci..

[99] Eimer M., Holmes A., McGlone F.P. (2003). The role of spatial attention in the processing of facial expression: An ERP study of rapid brain responses to six basic emotions. Cogn. Affect. Behav. Neurosci..

[100] Bamford S., Broyd S.J., Benikos N., Ward R., Wiersema J.R., Sonuga-Barke E. (2015). The late positive potential: A neural marker of the regulation of emotion-based approach-avoidance actions?. Biol. Psychol..

[101] Peeters G. (2002). From good and bad to can and must: Subjective necessity of acts associated with positively and negatively valued stimuli. Eur. J. Soc. Psychol..

[102] Klauer K.C., Teige-Mocigemba S., Spruyt A. (2009). Contrast effects in spontaneous evaluations: A psychophysical account. J. Personal. Soc. Psychol..

[103] Glaser J., Banaji M.R. (1999). When fair is foul and foul is fair: Reverse priming in automatic evaluation. J. Personal. Soc. Psychol..

[104] Moskowitz G.B., Ignarri C. (2009). Implicit volition and stereotype control. Eur. Rev. Soc. Psychol..

[105] Moskowitz G.B., Gollwitzer P.M., Wasel W., Schaal B. (1999). Preconscious control of stereotype activation through chronic egalitarian goals. J. Personal. Soc. Psychol..

[106] Moskowitz G.B. (2010). On the Control over Stereotype Activation and Stereotype Inhibition. Soc. Personal. Psychol. Compass.

[107] Moskowitz G.B., Salomon A.R., Taylor C.M. (2000). Preconsciously Controlling Stereotyping: Implicitly Activated Egalitarian Goals Prevent the Activation of Stereotypes. Soc. Cogn..

[108] Liu Y., Zhang D., Luo Y. (2015). How disgust facilitates avoidance: An ERP study on attention modulation by threats. Soc. Cogn. Affect. Neurosci..

[109] Zhang D., Liu Y., Wang L., Ai H., Luo Y. (2017). Mechanisms for attentional modulation by threatening emotions of fear, anger, and disgust. Cogn. Affect. Behav. Neurosci..

[110] Fiske S.T. (2012). Warmth and competence: Stereotype content issues for clinicians and researchers. Can. Psychol..

[111] Cuddy A.J.C., Glick P., Beninger A. (2011). The dynamics of warmth and competence judgments, and their outcomes in organizations. Res. Organ. Behav..

[112] Schul Y., Mayo R., Burnstein E. (2004). Encoding under trust and distrust: The spontaneous activation of incongruent cognitions. J. Personal. Soc. Psychol..

[113] Mayo R. (2015). Cognition is a matter of trust: Distrust tunes cognitive processes. Eur. Rev. Soc. Psychol..

[114] Posten A., Mussweiler T. (2013). When distrust frees your mind: The stereotype-reducing effects of distrust. J. Personal. Soc. Psychol..

[115] Abele A.E., Ellemers N., Fiske S.T., Koch A., Yzerbyt V. (2020). Navigating the social world: Toward an integrated framework for evaluating self, individuals, and groups. Psychol. Rev..

[116] Yzerbyt V., Corneille O. (2008). Cognitive Process: Reality Constraints and Integrity Concerns in Social Perception. On the Nature of Prejudice: Fifty Years after Allport.

[117] Abele A.E., Wojciszke B. (2007). Agency and communion from the perspective of self versus others. J. Personal. Soc. Psychol..

[118] Leach C.W., Ellemers N., Barreto M. (2007). Group virtue: The importance of morality (vs. competence and sociability) in the positive evaluation of in-groups. J. Personal. Soc. Psychol..

[119] Koch A., Imhoff R., Dotsch R., Unkelbach C., Alves H. (2016). The ABC of stereotypes about groups: Agency/socioeconomic success, conservative–progressive beliefs, and communion. J. Personal. Soc. Psychol..

